# FRET Measurement of Polymer Response under Shear

**DOI:** 10.3390/s21238033

**Published:** 2021-12-01

**Authors:** Ryo Iwao, Hiroki Yamaguchi, Makoto Obata, Yu Matsuda

**Affiliations:** 1 Department of Micro-Nano Mechanical Science and Engineering, Nagoya University, Furo-cho, Chikusa, Nagoya 464-8603, Japan; hiroki@nagoya-u.jp; 2 Interdisciplinary Graduate School of Medicine and Engineering, University of Yamanashi, 4-4-37 Takeda, Kofu 400-8510, Japan; mobata@yamanashi.ac.jp; 3 Department of Modern Mechanical Engineering, Waseda University, 3-4-1 Ookubo, Shinjuku-ku, Tokyo 169-8555, Japan

**Keywords:** fluorescence measurement, FRET (fluorescence resonance energy transfer), Couette flow, shear, polystyrene

## Abstract

Polymer solutions under shear flow are often observed in manufacturing processes. Classically, polymer behavior is represented by Kuhn’s bead-spring model, in which only the elongation of polymer chains is assumed. In recent years, the compression of polymer chains under shear flow has been reported. In this study, we investigated the behavior of polymer chains dissolved in various concentrations under shear flow. We measured the time variation of the fluorescence intensity emitted from a FRET (fluorescence resonance energy transfer) polymer, which enabled us to study the change in the distance between both ends of a polymer chain. The polymer chains appeared to stretch and compress depending on the concentration of the polymer solution. The results showed that the deformation of polymer chains was different from the classical theory. The FRET measurement is a promising diagnostic method for understanding the dynamics of polymer chains.

## 1. Introduction

The flow of a polymer solution has commonly appeared in synthetic and application processes for the manufacturing of various materials and fibers. This kind of flow is also commonly found in fluid control and other microfabricated sensing technologies [1,2]. Concerning the dynamics of polymer molecules under shear, they are known to be modeled by Kuhn’s bead-spring model, which deforms as a function of the shear rate and the angle of the molecule with respect to the shear direction when hydrodynamic force is applied [3]. The bead-spring model was improved as a tube model by introducing an elastic “spring constant” describing the overall restoring force that resists deformation [4]. When a polymer chain is in a steady-state flow, the hydrodynamic and elastic forces are in equilibrium. In these models, the hydrodynamic force is assumed to be an elongational force acting on the polymer chain. 

In semi-dilute polymer solutions, the deformation of polymer chains subjected to shear by a Couette flow was experimentally investigated by Dunston et al. [5,6]. In their studies, polymer molecules were found to align parallel to the shear direction at shear rates below 500 s^−1^ and align perpendicular to the shear at higher shear rates. These results were inconsistent with the classical assumption that polymer molecules are elongated when shear is applied [3,4]. Simulations focusing on a single polymer, which could be assumed as a limit of a dilute solution system, suggested that the buckling and stretching of polymer chains in solution were repeated under shear [1,7]. This result was also different from the classical assumption and was confirmed in experiments [8,9]. Although it was predicted that the response of polymers under shear varies with polymer concentration, to the best of the authors’ knowledge, there has been no systematic study of the change in polymer response to concentration using the same sample.

In this study, we investigated the response of polymer chains dissolved in various concentrations under shear induced by a Couette flow. For this purpose, we focused on fluorescence resonance energy transfer (FRET) molecules. A FRET molecule is composed of a pair of fluorescent molecules added to both ends of a polymer chain. FRET is a phenomenon of non-radiative energy transfer from a fluorescent molecule (donor) to another (acceptor). Since the efficiency of the energy transfer from the donor molecule to the acceptor molecule depends on their distance, we can derive the distance between fluorescent molecules at both ends of FRET molecules, i.e., the elongation and compression of FRET molecules, by observing the change in the fluorescence intensity of FRET molecules. Therefore, we employed a FRET molecule as a probe molecule in a polymer solution under shear flow.

## 2. FRET Molecule

To evaluate the elongation and compression of polymer chains under shear, a solution of FRET polymer and polystyrene (Sigma-Aldrich, St. Louis, MO, USA, $M_{w}$ = 35,000) in toluene solvent was prepared as a test fluid. The FRET polymer, Pyr-PSt-C343, was prepared by atom transfer radical polymerization (ATRP) using an initiator bearing pyrene followed by a click reaction at the terminating end with a propargyl group containing coumarin343. To afford a polymer sample with high end-group fidelity, ATRP is advantageous over reversible addition–fragmentation chain transfer (RAFT) polymerization, which requires an external radical initiator. In addition, ATRP affords a polymer-containing halogen at the terminating end, which is easily substituted by azide functionality and then is readily conjugated with terminal alkyne via click chemistry.

### 2.1. Principles of FRET

FRET occurs when the following three conditions are met: (1) the fluorescence spectrum of the donor molecule overlaps with the absorption spectrum of the acceptor molecule, (2) the donor and the acceptor molecule are in close proximity to each other, and (3) the orientation moments of the two molecules are in an appropriate orientation relationship. The energy efficiency E of FRET, which is the ratio of the energy transferred from the donor molecule to the acceptor molecule, is related to the distance r between the donor and acceptor molecules according to Förster’s equation [10,11]:

(1) $$\begin{array}{c} r={\left(\frac{1}{E}-1\right)}^{\frac{1}{6}}R_{0} \end{array}$$

where $R_{0}$ is the distance at which the energy efficiency of FRET is 50%. The energy efficiency E of FRET is expressed by the following equation using the quantum yield $\phi_{D}^{0}$ of the donor without the acceptor molecules and $\phi_{D}$ of the donor with the acceptor molecules [10,11].

(2) $$\begin{array}{c} E=1-\frac{\phi_{D}}{\phi_{D}^{0}} \end{array}$$

Furthermore, the quantum yield can be expressed in terms of the absorbance and fluorescence intensity as

(3) $$\begin{array}{c} E=1-\frac{A_{D}^{0}\left(\lambda_{D}\right)}{A_{D}\left(\lambda_{D}\right)}\cdot \frac{I_{D}\left(\lambda_{D},\lambda_{D}^{em}\right)}{I_{D}^{0}\left(\lambda_{D},\lambda_{D}^{em}\right)} \end{array}$$

where $A_{D}^{0}\left(\lambda_{D}\right)$ and $A_{D}\left(\lambda_{D}\right)$ are the absorbance without and with the acceptor molecules, and $I_{D}^{0}\left(\lambda_{D},\lambda_{D}^{em}\right)$ and $I_{D}\left(\lambda_{D},\lambda_{D}^{em}\right)$ represent the emission intensity without and with the acceptor molecules, respectively. $\lambda_{D}$ is the excitation wavelength of the donor molecule, and $\lambda_{D}^{em}$ is the emission wavelength of the donor molecule [10,11]. Therefore, by measuring the emission intensity of the donor molecule, one can evaluate the end-to-end distance of the polymer chain r. In this study, we measured the time variation of the fluorescence intensity of the donor molecule starting immediately after applying shear to evaluate the behavior of the end-to-end distance of the FRET polymer.

### 2.2. Synthesis of FRET Polymer

**Figure 1:**
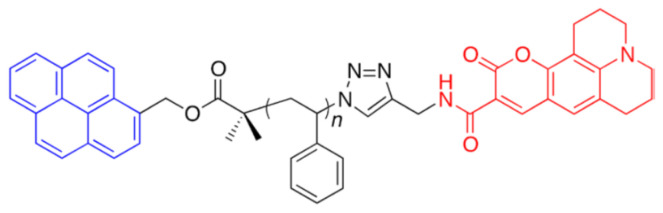
Chemical geometries of Pyr-PSt-C343.

**Figure 2:**
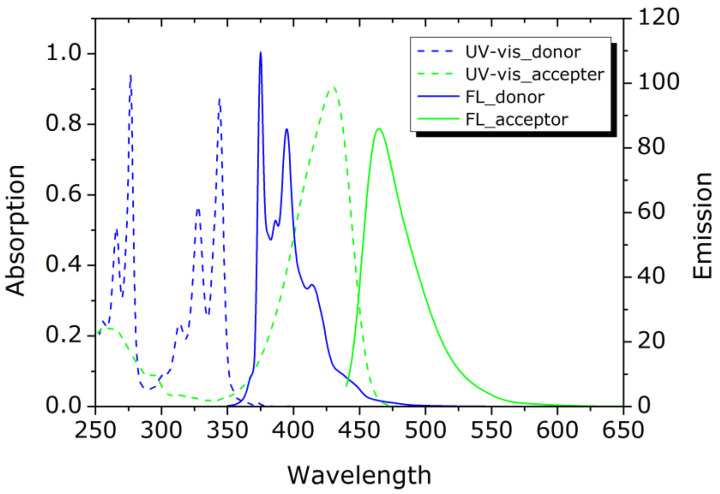
The emission spectrum of the donor molecule and the absorption spectrum of the acceptor of FRET polymer.

As the FRET polymer, we used Pyr-PSt-C343 (Figure 1), which is composed of polystyrene with pyrene as the donor molecule and coumarin343 as the acceptor molecule by addition polymerization at both ends. The emission spectrum of the donor molecule and the absorption spectrum of the acceptor molecule are shown in Figure 2. The overlap between the respective spectra indicates that the energy transition occurs when the donor molecule is excited.

#### 2.2.1. Preparation of ATRP Initiator Bearing FRET Donor

**Figure 3:**
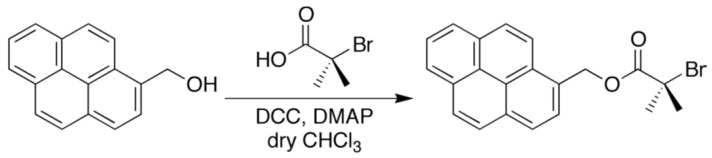
Synthesis of Pyr-BrB.

2-Bromoisobutylic acid (203.5 mg, 1.22 mmol), 1-pyrenemethanol (232.0 mg, 1.00 mmol), and 4-dimethylaminopyridine (12.1 mg, 0.10 mmol) were placed in a test tube and dissolved with dry CHCl_3_ (3 mL). A solution of *N*,*N*′-dicyclohexylcarbodimide (DCC; 309.3 mg, 1.50 mmol) in dry CHCl_3_ (2 mL) was added to this solution. The mixture was stirred at room temperature for 24 h under dark conditions. The resulting reaction mixture was diluted with CHCl_3_, washed with 1 M HCl (aq), sat. NaHCO_3_ (aq) and brine, and dried over Na_2_SO_4_. The crude product was purified by preparative gel permeation chromatography (GPC) to give Pyr-BrB (263 mg, 0.69 mmol) as shown in Figure 3.

#### 2.2.2. Preparation of Clickable FRET Acceptor

**Figure 4:**
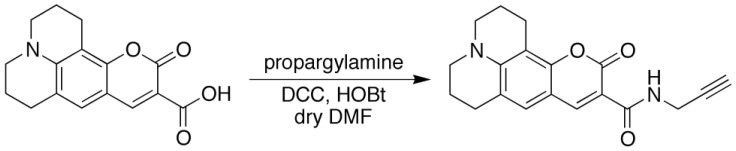
Synthesis of C343-pa.

Coumarin343 (142.4 mg, 0.50 mmol), propargylamine (117.1 mg, 2.13 mmol), and 1-hydroxybenzotriazole (HOBt; 107.0 mg, 0.79 mmol) were placed in a test tube and dissolved with dry DMF (2 mL). Then, a solution of DCC (207.5 mg, 1.01 mmol) in dry DMF (1 mL) was added to the solution, and the mixture was stirred at room temperature for 72 h. After removing the solvent, the crude product was purified by preparative GPC to afford C343-pa (55.6 mg, 0.17 mmol) as shown in Figure 4.

#### 2.2.3. Preparation of Polystyrene Bearing FRET Pair by ATRP-Click Approach

**Figure 5:**
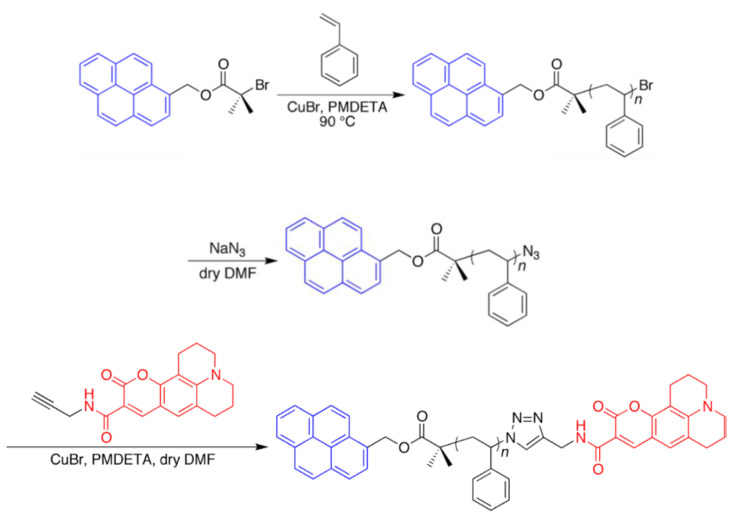
Synthesis of Pyr-PSt-C343 by ATRP-Click approach.

Styrene (1.04 g, 10 mmol), Pyr-BrB (38.4 mg, 0.10 mmol), and *N*,*N*,*N*′,*N*″,*N*″-pentamethyldiethylenetriamine (PMDETA; 17.4 mg, 0.10 mmol) were placed in a Schlenk tube and sealed with a rubber septum. The solution was degassed by five freeze–pump–thaw cycles. CuBr (14.1 mg, 0.10 mmol) was quickly added to the solution while the solution was frozen, and then the tube was evacuated and backfilled with N_2_ gas several times. The tube was immersed in an oil bath preheated at 90 °C to initiate polymerization. The polymerization was carried out for 6 h. The crude product was purified by reprecipitation from CHCl_3_–CH_3_OH to give Pyr-PSt-Br (562.7 mg; *M*_n_ = 7700, *M*_w_/*M*_n_ = 1.09). Pyr-PSt-Br (386.9 mg, 0.05 mmol), NaN_3_ (66.4 mg, 1.02 mmol), and dry DMF (2 mL) were placed in a test tube and sealed with a rubber septum. The solution was stirred at room temperature for 72 h. The solution was diluted with CHCl_3_, and then insoluble salt was filtered off. The filtrate was evaporated, and the crude product was purified by reprecipitation from CHCl_3_–CH_3_OH to give Pyr-PSt-N_3_ (375.5 mg; *M*_n_ = 7770, *M*_w_/*M*_n_ = 1.12). Pyr-PSt-N_3_ (310.9 mg, 0.04 mmol), C343-pa (26.1 mg, 0.08 mmol), PMDETA (10 μL), and dry DMF (1 mL) were placed in a Schlenk tube. The mixture was degassed by five freeze–pump–thaw cycles. CuBr (5.6 mg, 0.04 mmol) was quickly added to the solution while the solution was frozen, and then the tube was evacuated and backfilled with N_2_ gas several times. The mixture was stirred at 90 °C for 9 h. The crude product was purified by preparative GPC followed by reprecipitation from CHCl_3_–CH_3_OH to give Pyr-PSt-C343 (268.1 mg; *M*_n_ = 7420, *M*_w_/*M*_n_ = 1.07) (Figure 5). The extent of coumarin343 dye introduction at the terminating end was estimated to be 86.7% using UV-vis spectroscopy. Detailed information about Pyr-PSt-C343, such as molecular spectra, can be found in Supplementary Materials.

## 3. Experimental Methods

**Figure 6:**
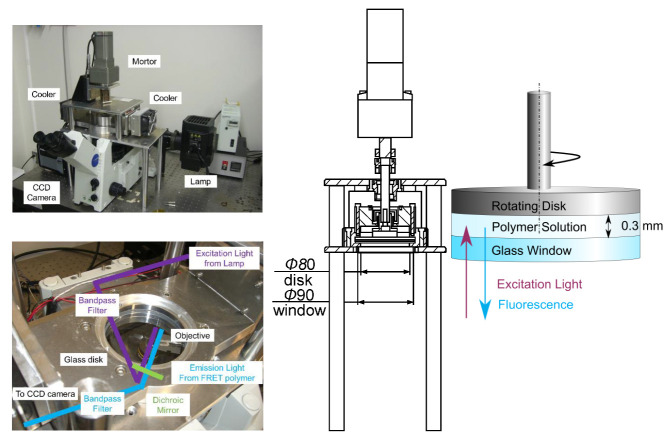
Schematics of the experimental setup.

In this study, we applied shear to a polymer solution and measured the fluorescence intensity of the FRET polymer in the polymer solution. Schematics of the experimental setup are shown in Figure 6. The excitation light emitted from a Xenon short-arc lamp (U-LH75XEAPO, Olympus, Tokyo, Japan) passed through a long-wavelength cut filter (FF01-336/19-25, Semrock, IDEX Corporation, Lake Forest, IL, USA) was reflected by a dichroic mirror (Di01-R355-25x36, Semrock, IDEX Corporation, Lake Forest, IL, USA) and passed through an objective lens (MPLFLN10x, Olympus). The FRET polymer in the polymer solution was excited by the excitation light, and the emitted light, which passed through the objective lens, dichroic mirror, and bandpass filter, was detected by an EMCCD camera (ProEM 1024B, Teledyne Princeton Instruments, Trenton, NJ, USA). The polymer solution was fed into the gap between a fixed glass window (radius *R* = 45 mm) and a rotating disk (radius *r* = 40 mm). The rotating disk was moved upwards by 0.3 mm from the point of contact with the glass surface to make the gap. The rotating disk was rotated by a motor (NX940AS-PS5-1, Oriental Motor, Tokyo, Japan) to apply shear to the polymer solution. When the polymer solution was sheared, heat was generated due to viscous dissipation and friction, and the temperature of the solution would increase. To maintain the temperature, a cooling system (FPH1-12708AC, Z-Max, Tokyo, Japan) was installed, and the temperature of the polymer solution was controlled to 295 K. Since toluene, which was the solvent used for the polymer solution, is volatile, a solvent trap with a phosphate buffer solution was installed. The rotating disk was operated for 2 h before the measurements to ensure that the measurement was carried out with the toluene vapor saturated. The fluorescence images were taken every minute with an exposure time of 60 s, starting from the actuation of the rotating disk.

The critical overlap concentration $c^{*}$, which is the standard for the concentration of the solution, was calculated as follows: The Flory radius $R_{F}$ of polystyrene in a good solvent is calculated as [4]

(4) $$\begin{array}{c} R_{F}=bN^{\nu } \end{array}$$

Here, b is the Kuhn segment length, N is the number of segments, and ν is the swelling exponent. We adopted the length (b = 0.25 nm), calculated by assuming that the polystyrene backbone of polystyrene was all-trans conformal [12]. N was calculated as the degree of polymerization of the polystyrene molecule, N≈ 337. The swelling exponent for a good solvent is ν=0.588 [13]. The critical overlap concentration $c^{*}$ in a good solvent is expressed by the following equation using molecular weight $M_{w}$, Flory radius $R_{F}$, and Avogadro’s constant $N_{A}$ [4]

(5) $$\begin{array}{c} c^{*}=\frac{M_{w}}{N_{A}R_{F}{}^{3}} \end{array}$$

This gave the critical overlap concentration of polystyrene ($M_{w}$ = 35,000) in the toluene solvent as $c^{*}=0.13\;g/{\mathrm{cm}}^{3}$. 

The critical overlap concentration $c^{*}$ is also defined as follows [14]:

(6) $$\begin{array}{c} c^{*}=\frac{2.5}{\left[\eta \right]} \end{array}$$

Since the dependence of the intrinsic viscosity [η] on the molecular weight can be represented by the Mark–Houwink equation,

(7) $$\begin{array}{c} \left[\eta \right]=KM^{\alpha } \end{array}$$

Here, K and α are the Mark–Houwink parameters, and they depend on the polymer-solvent system; for the polystyrene-toluene solutions, K=0.00862, α=0.736 [14]. The critical overlap concentration calculated from Equations (6) and (7) was $c^{*}=0.13\;g/{\mathrm{cm}}^{3}$. 

Since the actual value of the polystyrene used in the measurement was unknown, and it was necessary to assume each value, ambiguity existed in the calculation of the critical concentration because assumptions must be made for each value. However, the values of the critical concentration by the two calculation methods were, surprisingly, in good agreement. We used these values to calculate the concentration hereafter. 

The concentration of the FRET polymer was determined to be the distance between the polymers having a FRET efficiency of 0.1%. To find the maximum concentration without FRET between different FRET molecules, we considered a close-packed sphere of radius $R=R_{g}$ + r/2 and calculated on a face-centered cubic lattice. The radius of gyration $R_{g}$ of polystyrene with $M_{w}$ = 7940 in toluene was $R_{g}$ = 2.8 nm from the following equation [15]:

(8) $$\begin{array}{c} R_{g}=\left(0.0150\right)M_{w}{}^{0.583\pm 0.022}\times 10^{-9}\;\left[m\right] \end{array}$$

The distance r at which the FRET efficiency was 0.1% was 11.4 nm from the Equation (1). As a result, the molar concentration at the closest packing was 4.8 × 10^−7^ mol/mL, which was used as the upper limit of the FRET polymer concentration.

## 4. Results and Discussion

**Figure 7:**
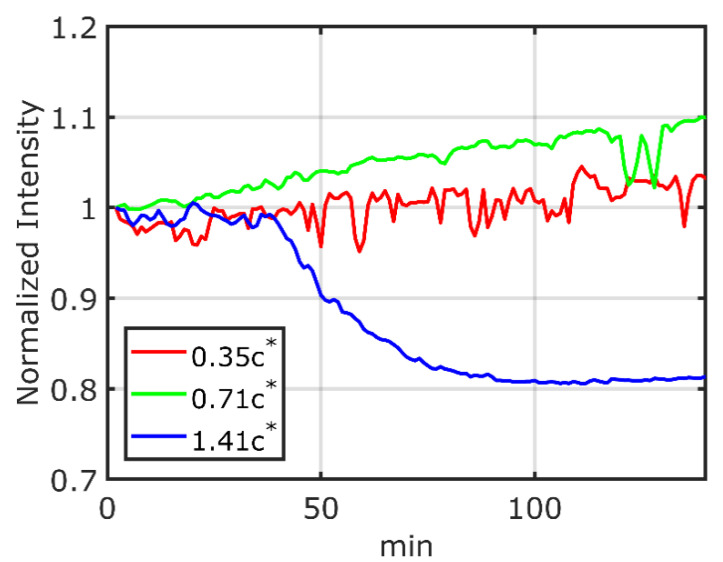
The time variations of the normalized fluorescence intensities of FRET molecules in polymer solutions with concentrations of 0.35 $c^{*}$, 0.71 $c^{*}$, and 1.41 $c^{*}$ at a shear rate of 333 $s^{-1}$.

Figure 7 shows the time variation of the fluorescence intensity from the FRET molecules in the polymer solutions with concentrations of 0.35 $c^{*}$, 0.71 $c^{*}$, and 1.41 $c^{*}$ at a shear rate of 333 s^−1^. The fluorescence intensities are normalized using the fluorescence intensity *I*_0_ taken 2 min after the start of the actuation of the rotating disk to account for variations in the concentration of the FRET molecules. In the 0.35 $c^{*}$ and 0.71 $c^{*}$ polymer solutions, the fluorescence intensities show a tendency to increase with time. On the other hand, in the 1.41 $c^{*}$ polymer solution, the fluorescence intensity tends to decrease with time. These changes in the fluorescence intensity can be attributed to the change in FRET efficiency, that is, it can be interpreted as the change in the end-to-end distance of the FRET molecules in the polymer solution. Lower intensity indicates compression, and higher intensity indicates elongation. Therefore, when a shear force is applied, the FRET molecules are observed to elongate or compress, depending on the concentration of the polymer solution. However, since there is ambiguity in the value of the critical overlap concentration $c^{*}$, it is not possible to determine the exact state of the solution according to the concentration.

The reason for the observed compression of the FRET molecules at 1.41 $c^{*}$ could be attributed to the fact that the FRET molecules are restricted by the presence of the surrounding polymer molecules. Meanwhile, the elongation of the FRET molecules at 0.35 $c^{*}$ and 0.71 $c^{*}$ is considered to be due to the existence of unrestricted space around the FRET molecules for free deformation. The higher elongation under shear at 0.71 $c^{*}$ than at 0.35 $c^{*}$ is shown in Figure 7, and the reason for this tendency could be explained because the hydrodynamic force under shear is calculated by the bead-spring dumbbell model as follows [3]:

(9) $$\begin{array}{c} f_{\mathrm{hyd}}\left(R\right)=6\pi \eta x\dot{\gamma }R\sin2\theta \end{array}$$

where *η* is the viscosity for a bead of radius x, $\dot{\gamma }$ is the shear rate, R is the distance between beads, and θ is the angle taken by the beads in the direction of shear [3]. Since the viscosity of the 0.71 $c^{*}$ polymer solution is higher than that of the 0.35 $c^{*}$ solution, the Equation (6) suggests that the hydrodynamic effect on the FRET molecule itself is larger for 0.71 $c^{*}$ than that for 0.35 $c^{*}$. In addition, since the distance between both ends of the molecule before shearing is longer in less concentrated polymer solutions [16], the initial values of the end-to-end distance between the ends among the solutions of different concentrations may be different, which affect the normalized intensities in Figure 7. 

In Figure 7, 1.41 $c^{*}$ shows that the relaxation time of this system is in the order of several tens of minutes, which is much longer than that of the polymer chain itself. The maximum relaxation time of polymethyl methacrylate with a molecular weight of 200 kg/mol was reported to be 0.104 ms at a mass concentration of 20% (3.3 $c^{*}$) and 0.207 ms at 28% (4.6 $c^{*}$) [17]. In observing the structure of hydroxypropyl guar gels formed under shear using atomic force microscopy, molecular rearrangement with compression called “balling” was reported [18]. This result suggests that the polymer chain itself is not only compressed but also undergoes a large structural change, such as the “balling” by the application of shear, which could largely increase the relaxation time. 

In a similar FRET experiment in a Couette flow, a decrease in the fluorescence intensity was reported immediately after the application of shear [17], while in this study, a decrease in the fluorescence intensity was observed after a certain period of time from the start of applying shear in a solution of 1.41 $c^{*}$ concentration. The relaxation time of the fluorescence intensity is also different, about 60 min in this study compared to about 10 min in the previous experiment [17]. These differences might be from the difference in the measurement conditions; the previous experiments were performed at higher shear rates of 616 $s^{-1}$ to 1848 $s^{-1}$ compared to the shear rate of 333 $s^{-1}$ in this study, which indicates that the time required for rearrangement in this study is longer than in the previous experiments. This may lead to the difference in the delay and the relaxation times.

## 5. Conclusions

The deformation of the polymer chain occurs in a shear flow. The behavior of the deformation depends on the concentration of the polymer solution. By measuring the fluorescent intensity of the FRET molecules in a Couette flow, different behaviors of elongation and compression were observed for different concentrations of polymer solutions. We successfully measured the time variation of the fluorescence intensity of the FRET molecules under shear. The different behaviors depending on concentration were observed. As the concentration increased, the increasing fluorescence intensity turned to a decreasing trend with time. However, the critical concentration of the polymer solution was calculated with a large range, and there remained some ambiguity in the state of the polymer solution. The detailed data on the properties of the polymer are highly necessary for further detailed analysis.

The time variation of the deformation of the polymer showed that the changes occurred on a timescale larger than the relaxation time of the polymer chain itself. It was considered that the application of shear caused rearrangement of the molecules in the polymer solution. Not only was polymer elongation as predicted by the classical theory observed, but also polymer compression. Experiments on the same sample with different concentrations will help us understand the mechanism of polymer behavior under shear. 

## Data Availability

The data presented in this study are available on request from the corresponding author.

## Supplementary Materials

The following are available online at https://www.mdpi.com/article/10.3390/s21238033/s1. Section S1: Analytical Methods of Materials, Figure S1: ^1^H NMR spectrum of Pyr-BrB in CDCl_3_, Figure S2: ^1^H NMR spectrum of C343-pa in CDCl_3_, Figure S3: GPC traces of Pyr-PSt-Br, Pyr-PSt-N_3_ and Pyr-PSt-C343, Figure S4: UV-vis and fluorescence spectra of Pyr-BrB in THF, Figure S5: UV-vis and fluorescence spectra of C343-pa in THF, Figure S6: UV-vis spectrum of Pyr-PSt-C343 in THF.
