# Supplementary material for: FRET Measurement of Polymer Response under Shear
Source: Sensors (Basel). 2021 Dec 1;21(23):8033. doi: 10.3390/s21238033 (PMC8659492; doi:10.3390/s21238033)
Supplement: Supplementary file 1 [file sensors-21-08033-s001.zip › sensors-1476482-supplementary.pdf]

Supplementary Materials

FRET Measurement of Polymer Response Under Shear

Ryo Iwao[1,*], Hiroki Yamaguchi[1], Makoto Obata[2] and Yu Matsuda [3,*]

[1] Department of Micro-Nano Mechanical Science and Engineering, Nagoya University, Furo-cho, Chikusa, Nagoya, Aichi, 464-8603, Japan; iwao.riyou@j.mbox.nagoya-u.ac.jp (R.I.); hiroki@nagoya-u.jp (H.Y.)
[2] Interdisciplinary Graduate School of Medicine and Engineering, University of Yamanashi, 4-4-37 Takeda, Kofu, Yamanashi 400-8510, Japan; mobata@yamanashi.ac.jp
[3] Department of Modern Mechanical Engineering, Waseda University, 3-4-1 Ookubo, Shinjuku-ku, Tokyo, 169-8555, Japan; y.matsuda@waseda.jp
* Correspondence: iwao.riyou@j.mbox.nagoya-u.ac.jp; y.matsuda@waseda.jp

Section S1. Analytical Methods of Materials

^{1}H NMR spectra were recorded using the AVANCE 400 (400 MHz, Bruker, Biospin K.K. Yokohama, Japan) instruments. Ultraviolet-visible (UV-vis) spectra were recorded on a V-550 spectrophotometer (JASCO Co. Tokyo, Japan). Fluorescence spectra were recorded on an FP-6300 spectrofluorometer (JASCO Co. Tokyo, Japan). Gel permeation chromatography (GPC) was performed with an HPLC system (pump, LC-20AT; refractive index detector, RID-10A, Shimadzu Co.) using Styragel HR4 (7.8 × 300 mm) (Waters, MA), Styragel HR3 (7.8 × 300 mm), and Styragel HR1 (7.8 × 300 mm) columns as a stationary phase and tetrahydrofuran (THF) as a mobile phase at a flow rate of 1 mL min^{-1}. The GPC system was calibrated with 11 polystyrene standards (Showa Denko, K.K., Tokyo, Japan) ranging in molecular mass from 1.31 to 2210 kg mol^{-1}. The number average molecular mass (M_n) and dispersity (M_w/M_n) were calculated by the polystyrene calibration.

Figure S1 ^{1}H NMR spectrum of Pyr-BrB in $CDCl_3$
Figure S2 ^{1}H NMR spectrum of C343-pa in $CDCl_3$
Figure S3 GPC traces of Pyr-PSt-Br, Pyr-PSt-N_3 and Pyr-PSt-C343
Figure S4 UV-vis and fluorescence spectra of Pyr-BrB in THF
Figure S5 UV-vis and fluorescence spectra of C343-pa in THF
Figure S6 UV-vis spectrum of Pyr-PSt-C343 in THF

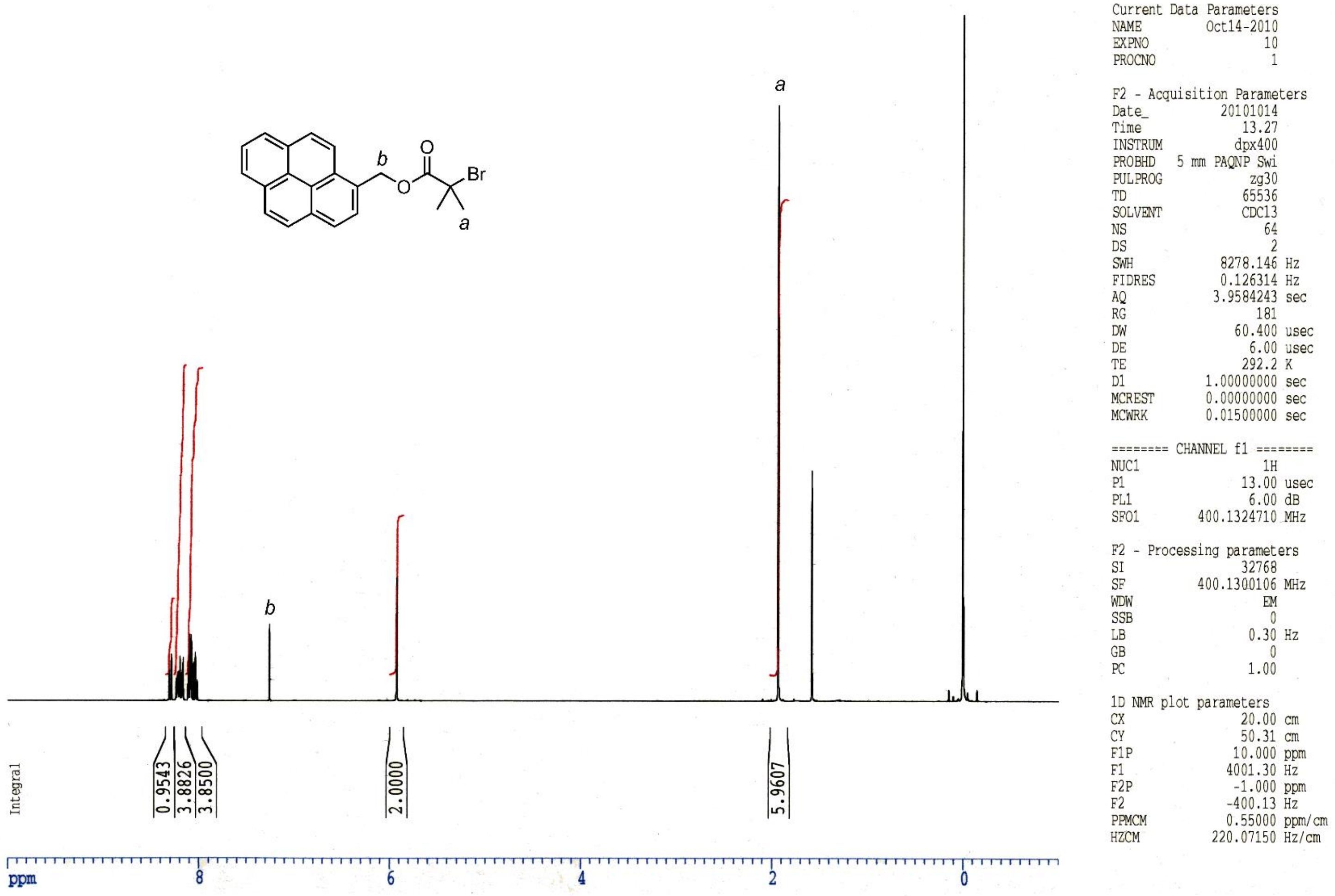

Figure S1 ^{1}H NMR spectrum of Pyr-BrB in $CDCl_3$

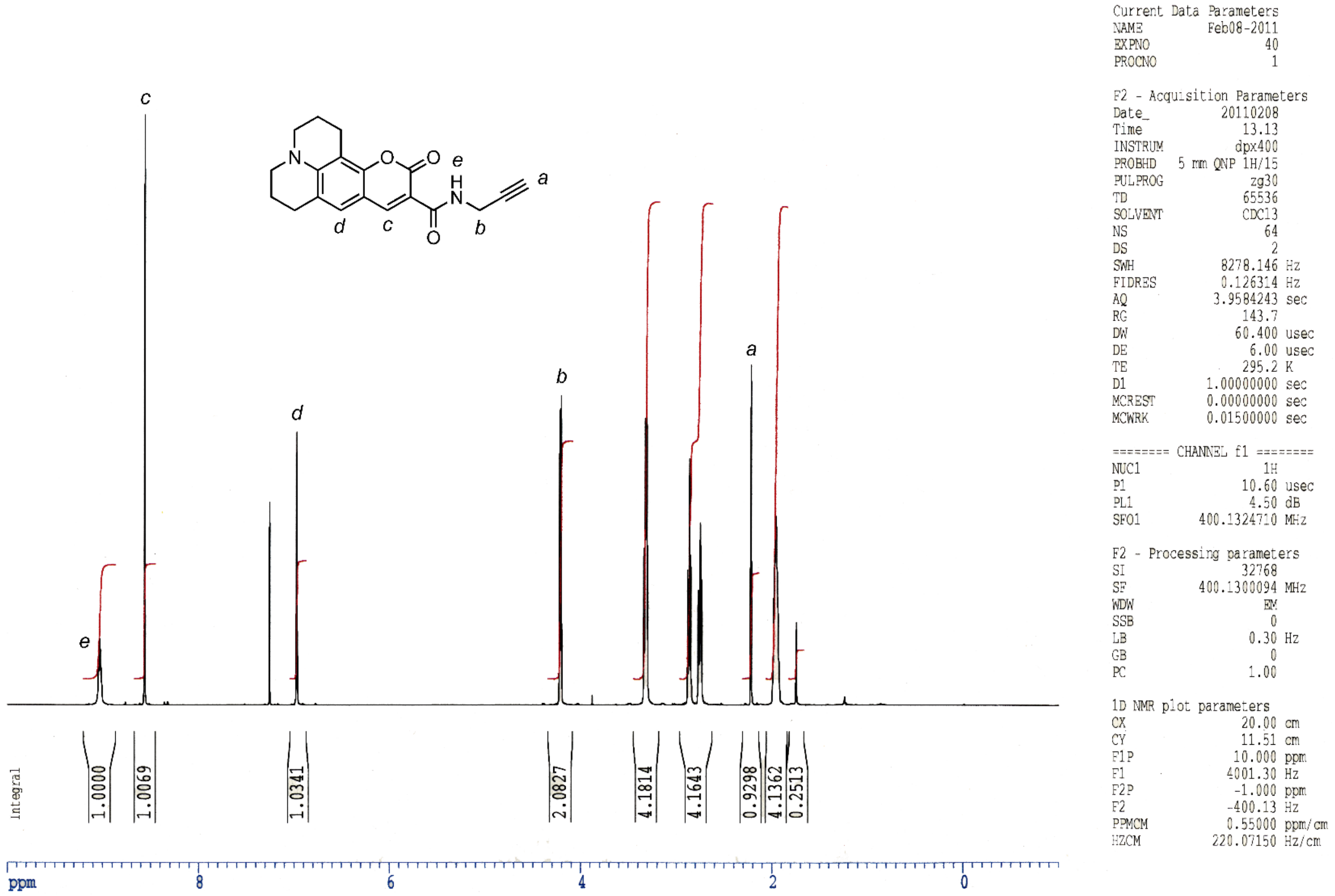

Figure S2 ^{1}H NMR spectrum of C343-pa in $CDCl_3$

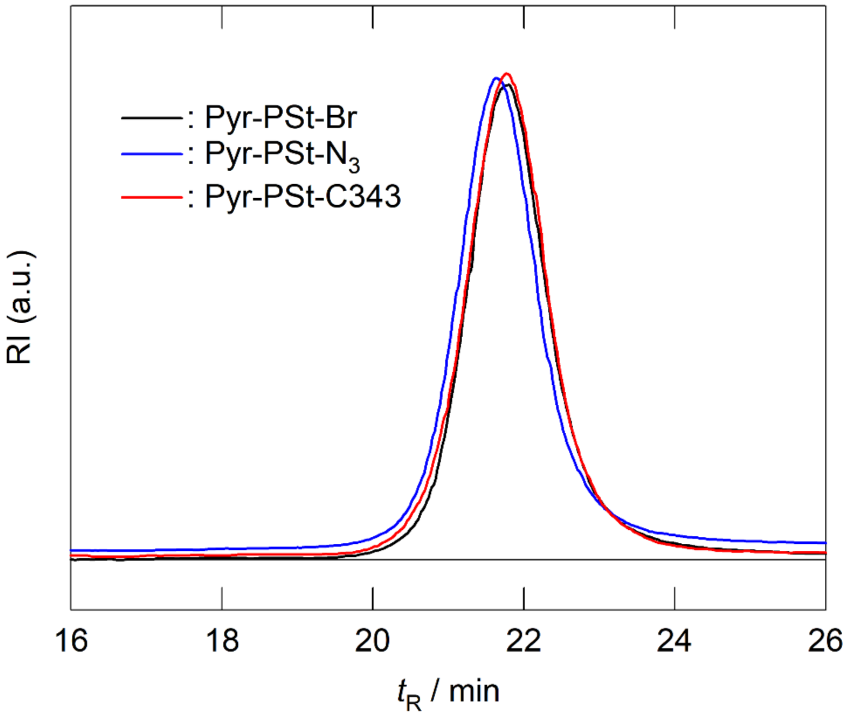

Figure S3 GPC traces of Pyr-PSt-Br (black), Pyr-PSt-N_3 (blue), and Pyr-PSt-C343 (red).

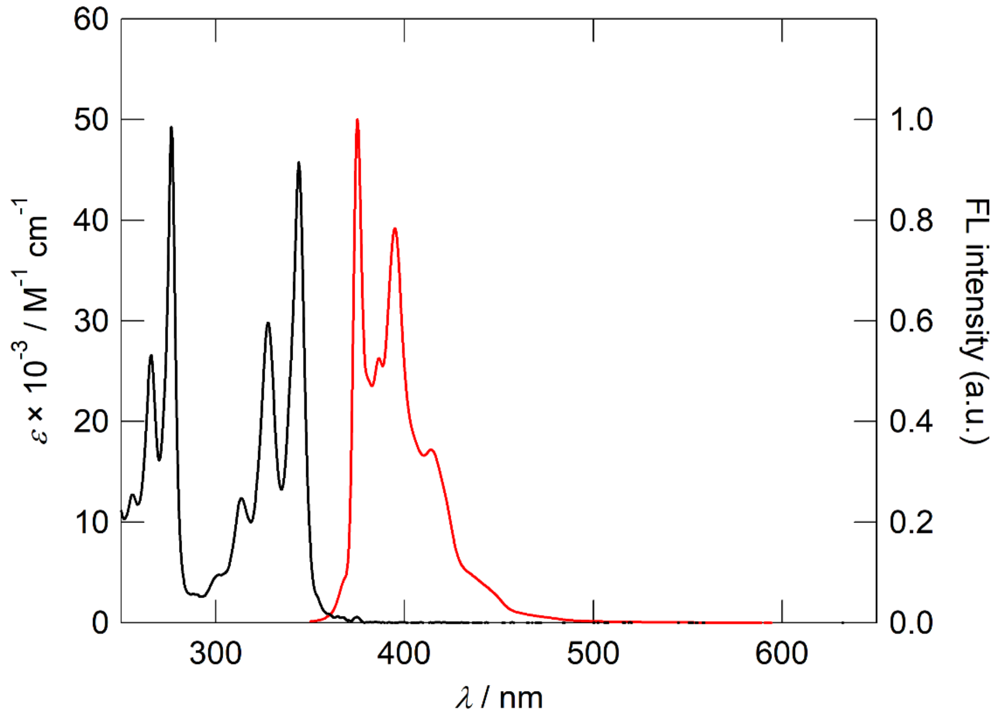

Figure S4 UV-vis and fluorescence spectra of Pyr-BrB in THF. [Pyr-BrB] = 1.91 × 10^{-5} M.

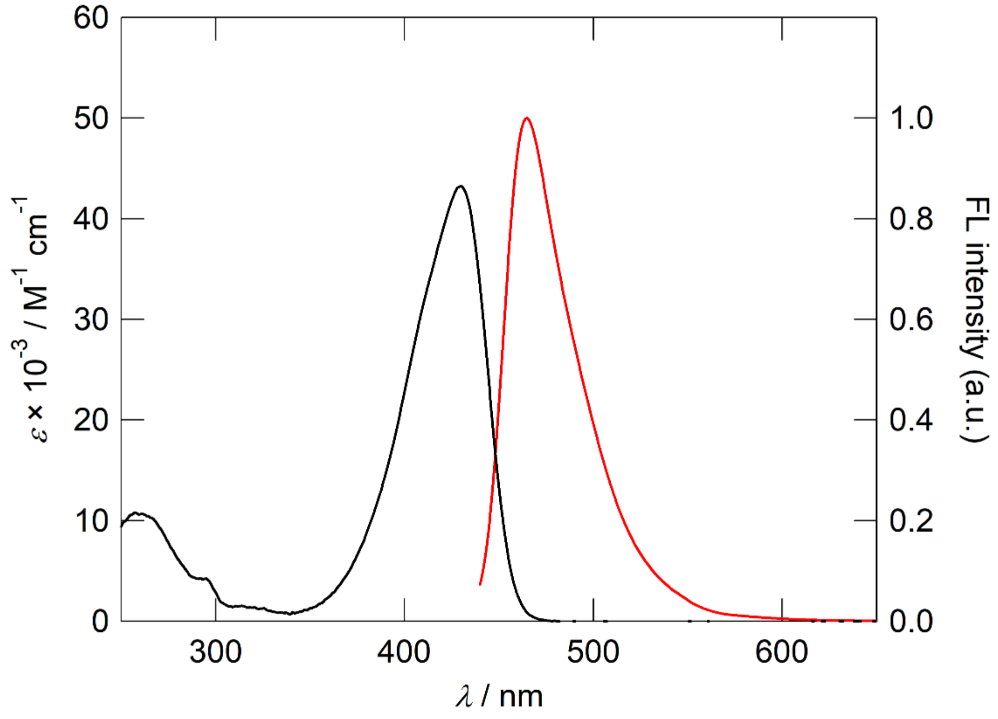

Figure S5 UV-vis and fluorescence spectra of C343-pa in THF. [C343-pa] = 2.10 × 10^{-5} M.

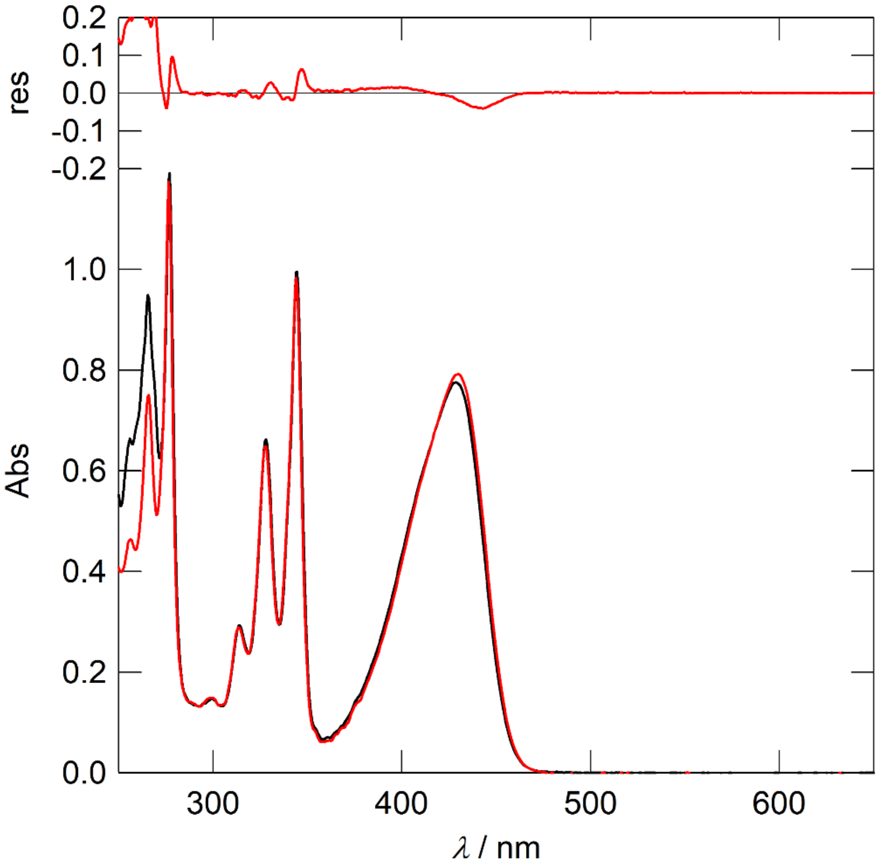

Figure S6 UV-vis spectrum of Pyr-PSt-C343 in THF (black line) and best-fitted line (red line; A_{calc}) by superposition of UV-vis spectra of Pyr-BrB and C343-pa. [Pyr-PSt-C343] = 0.156 mg mL^{-1}.

Estimation of the degree of the end-group modification

The superimposed spectrum was calculated as follows

$$A_{calc} = \varepsilon_{Pyr-BrB}[Pyr - BrB] + \varepsilon_{C343-pa}[C343 - pa] \quad (S1)$$

in which $\varepsilon_{Pyr\text{-}BrB}$ and $\varepsilon_{C343\text{-}pa}$ are molar absorption coefficients, respectively, at a given wavelength. The UV-vis spectrum of Pyr-PSt-C343 was fitted with Equation (S1) in the region from 300 to 500 nm to give molar concentrations [Pyr-BrB] and [C343-pa] as 2.11 × 10^{-5} and 1.83 × 10^{-5} M, respectively. Hence the degree of end-group modification was estimated to be 86.7 %.

Fidelity of the initiating end

On the assumption of the presence of pyrene at the initiating end, number-average molecular mass can be estimated from the above UV-vis spectrum (Figure S6) as follows;

$$M_{n,UV} = \frac{0.156}{2.11 \times 10^{-5}} = 7393 \quad (S2)$$

This value is reasonably close to the value estimated by GPC, indicating good conservation of pyrene dye at the initiating end.
